# Spatial Heterogeneity as a Genetic Mixing Mechanism in Highly Philopatric Colonial Seabirds

**DOI:** 10.1371/journal.pone.0117981

**Published:** 2015-02-13

**Authors:** Robin Cristofari, Emiliano Trucchi, Jason D. Whittington, Stéphanie Vigetta, Hélène Gachot-Neveu, Nils Christian Stenseth, Yvon Le Maho, Céline Le Bohec

**Affiliations:** 1 Université de Strasbourg (UdS), Institut Pluridisciplinaire Hubert Curien, Laboratoire International Associé LIA-647 BioSensib (CSM-CNRS-UdS), Strasbourg Cedex 02, France; 2 Centre National de la Recherche Scientifique (CNRS), UMR 7178, LIA-647 BioSensib, Strasbourg Cedex 02, France; 3 Centre Scientifique de Monaco (CSM), LIA-647 BioSensib, 8, Quai Antoine 1er, Monaco, Principality of Monaco; 4 University of Oslo, Centre for Ecological and Evolutionary Synthesis, Department of Biosciences, Postboks 1066, Blindern, Oslo, Norway; Biodiversity Insitute of Ontario - University of Guelph, CANADA

## Abstract

How genetic diversity is maintained in philopatric colonial systems remains unclear, and understanding the dynamic balance of philopatry and dispersal at all spatial scales is essential to the study of the evolution of coloniality. In the King penguin, *Aptenodytes patagonicus*, return rates of post-fledging chicks to their natal sub-colony are remarkably high. Empirical studies have shown that adults return year after year to their previous breeding territories within a radius of a few meters. Yet, little reliable data are available on intra- and inter-colonial dispersal in this species. Here, we present the first fine-scale study of the genetic structure in a king penguin colony in the Crozet Archipelago. Samples were collected from individual chicks and analysed at 8 microsatellite loci. Precise geolocation data of hatching sites and selective pressures associated with habitat features were recorded for all sampling locations. We found that despite strong natal and breeding site fidelity, king penguins retain a high degree of panmixia and genetic diversity. Yet, genetic structure appears markedly heterogeneous across the colony, with higher-than-expected inbreeding levels, and local inbreeding and relatedness hotspots that overlap predicted higher-quality nesting locations. This points towards heterogeneous population structure at the sub-colony level, in which fine-scale environmental features drive local philopatric behaviour, while lower-quality patches may act as genetic mixing mechanisms at the colony level. These findings show how a lack of global genetic structuring can emerge from small-scale heterogeneity in ecological parameters, as opposed to the classical model of homogeneous dispersal. Our results also emphasize the importance of sampling design for estimation of population parameters in colonial seabirds, as at high spatial resolution, basic genetic features are shown to be location-dependent. Finally, this study stresses the importance of understanding intra-colonial dispersal and genetic mixing mechanisms in order to better estimate species-wide gene flows and population dynamics.

## Introduction

How colonial systems are maintained in non-cooperative species remains an important question in evolutionary biology [1,2]. Philopatric behaviour is usually considered to be the basis of coloniality [2]. It is thought to offer several selective advantages, such as a good knowledge of the higher quality breeding spots and of the pool of breeding partners linked to these spots [3–5], and it favours the selective value of proximal defensive behaviour [6] or allofeeding [7] through kinship selection. However, the drawback of such behaviour is the fragmentation of genetic diversity at the colony level [8], which leads to an increase in inbreeding within clusters of closely-related individuals, in turn potentially leading to local inbreeding depression [9,10]. Dispersal is therefore an essential balancing force in the conservation of colonial systems [2,11–13]. It is known to promote gene flow [14] and genetic adaptability [2] as well as plasticity in response to habitat changes [13,15].

Both philopatry and dispersal occur at all spatial scales in many colonial species [16,17], yet it has often been assumed that strong philopatric behaviour is a common trait of most pelagic seabirds [18–20]. Indeed, the benefits of philopatry and coloniality [21], the barriers to dispersal [22] and the dynamics of colony formation and extinction [23] have traditionally been explored under the hypothesis that philopatry is a default behaviour. Thus, several studies have documented a lack of genetic structure amongst distant colonies, which, in this context, seemed incompatible with assumed philopatry (the so-called “seabird paradox” of Milot *et al*. [24]).

It is often considered that the relatively weak evidence for structure, together with the high level of genetic diversity observed in many seabird species (e.g. [25–27]), are mostly the consequence of the random dispersal of a few juveniles [26,28]. On the other hand, the importance of intra-colonial dispersal in fitness enhancement has been studied through direct observations [28], and dispersal distance, even at very fine scale, has been shown to depend on species-specific ecological dynamics [8,17]. However, strict correlation between dispersal, site fidelity, and population structure has been questioned [29], and the importance of intra-colonial movements on genetic mixing has received comparatively little attention.

The King penguin, *Aptenodytes patagonicus*, which breeds in large colonies all around the sub-Antarctic area, is an ideal model to investigate these aspects of coloniality. It is known to choose and defend a breeding territory within the colony, even though it does not build a nest [30,31]. Breeders of this species have been described as having a marked philopatric behaviour across reproductive seasons and have been observed to use the same breeding site, within a radius of a few meters, year after year within their colony [30,31]. Return rates of juveniles to their natal colony are also typically high [32,33]. However, it is not yet clear whether natal philopatric behaviour also extends to the specific birth site within the colony, and whether this behaviour may result in local structuring of populations. In order to test this, a genetic study was conducted in the “Baie du Marin” colony on Possession Island, Crozet Archipelago. If philopatric behaviour indeed extends to breeding territory selection, it is expected that the colony will have a non-random genetic structure, with closely related individuals distributed in spatial clusters, as has already been observed in several non-avian philopatric species (e.g. [34,35]). However, even in known philopatric species, detection of genetic structure can be hindered by a variety of behaviours, such as juvenile and adult dispersion or extra-pair paternity [36]; therefore, the methods used to study it must be sensitive enough to detect even a weak signal [37].

When considering the genetic structure of a species, expected patterns may be analysed according to *(i)* global homogeneous processes or *(ii)* local heterogeneous processes whose effect varies across the area [38]. *(i)* The predictions associated with strong and consistent philopatric behaviour belong to the former class. Without taking spatial distribution of individuals into account, a skew in global population inbreeding descriptors may be expected [39], with an excess of homozygosity reflecting higher-than-random relatedness between paired individuals. However, a prior to F-statistics is that populations are in a state of equilibrium, which may be in contradiction with fine spatial and temporal scale analysis, such in our study [40,41]. Alternatively spatial autocorrelation should be detected at the colony level, reflecting a gradual isolation-by-distance trend, though this should only hold true if the process leading to spatial structure is homogenous at the individual level [38]. *(ii)* If, on the other hand, philopatric behaviour is a more variable trait dependent on local ecological conditions, the processes involved in colony structure are expected to belong to the second class. Local heterogeneous signatures should therefore be found, such as divergent population parameters for different areas of the colony [37]. Although inbreeding is not in itself an indicator of spatial structure, higher-than-expected individual inbreeding levels may reveal a non-random mating system, favouring relatives as potential partners. In such cases, a spatially-correlated distribution of inbreeding suggests a space-driven mating system [42,43]. Pairwise relatedness, calculated across spatially-defined windows, is another insight into the same phenomenon [42,44].

In this study, we use a combined approach including genetic, spatial and local ecological data to investigate the mechanisms of genetic mixing inside a seabird colony. We focus in particular on how fine-scale habitat heterogeneity patterns may influence local dispersal behaviour, and how lack of genetic structuring can emerge from small-scale heterogeneity in ecological parameters.

## Materials and Methods

### Permits and ethics statement

All animals in this study were handled only once in order to mark them with an external plastic pin tag (Floytag(r)) and to conduct morphological measurements. All procedures employed during this field work were approved by the Ethical Committee of the French Polar Institute (Institut Paul Emile Victor—IPEV) and conducted in accordance with its guidelines, also complying with French laws including those relating to conservation and welfare. Authorizations to enter the breeding site (permits n°2009–57 issued on August 26th 2009) and handle birds (permits n°2009–59 issued on August 29th 2009) were delivered first by the French “Ministère de l’Aménagement du Territoire et de l’Environnement” and then by the Terres Australes et Antarctiques Françaises (TAAF).

Handled animals were removed from the colony in order to minimize the disturbance to neighbouring birds and taken to a shelter a few meters away for manipulation. They were hooded to reduce their stress and manipulations lasted between 5 and 10 minutes. All blood-sampling and tagging material was sterilized (either sealed, or through chemical sterilization). Moreover, Vétédine soap and alcoholic antiseptic solutions were used to disinfect the skin before bleeding and tagging. Flesh wounds did not seem infected thereafter (personal observations on a subset of recaptured birds).

### Study site and sampling

**Fig 1:**
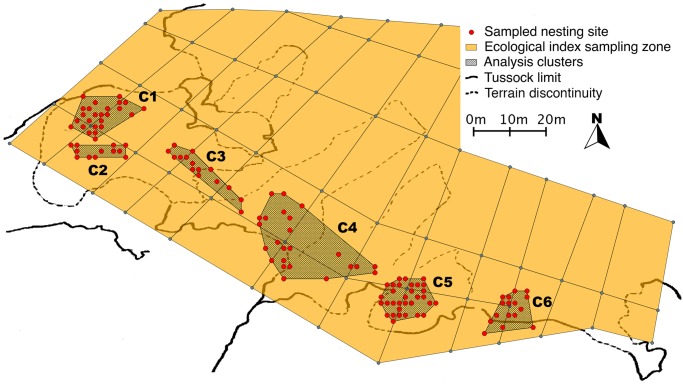
Sampling distribution. Sampling was restricted to the periphery of the colony. Orange zone boundaries are marked on the ground for remote parameter assessment. Shaded clusters run from C1 (north-west) to C6 (south-east). Orange zone: extent of the colony at the peak of the breeding season.

Our study was conducted during the summer season 2009/2010 in the king penguin colony of “La Baie du Marin”, on Possession Island, Crozet Archipelago (46°25’S, 51°45’E), which comprises around 16,000 breeding pairs [45]. Chicks born in a sub-section of the colony comprising approximately 10,000 breeding pairs (Antavia, [46]) were randomly selected and captured along a peripheral 120m-transect, in order to maximise separation distance. To minimize disturbance of breeding penguins, sampling was conducted no deeper than 4–5 nests towards the centre of the colony. Sampling was conducted at a high spatial resolution (~2 m between successive samples, i.e. every second nest at the time of sampling) in order to assess fine-scale genetic structure of the colony (sampling map is given in Fig. 1). The sampling area was partitioned according to natural terrain discontinuities (such as tussocks, rocks, or flooded patches), in a total of 6 clusters encompassing all sampled nests (Fig. 1). 175 early-hatched chicks were thus sampled during early brooding (~ 2 week old chicks) and temporarily tagged with a small external plastic pin (Floytag(r)). Each chick’s hatching site was geolocated with a Global Positioning System device (Garmin eTrex(r)), and further confirmed using visual grid markings in the field (*ca*. 10×10m cells). King penguins are monogamous during a reproductive season and lay a single egg each year [31,47], thus we exclude the possibility of full siblings in the study population. Blood samples (~ 100 μ L) were collected from the brachial veins using heparin-coated tubes, transferred to a Whatman(r) filter paper, dried, and later frozen at -20°C for the genetic analysis.

### DNA extraction, PCR and genotyping

DNA was extracted using a DNeasy(r) Blood and Tissue Kit (Qiagen) with a modified protocol. A prolonged initial incubation of the impregnated Whatman(r) paper in PBS buffer (24 h) was used to dissolve dried blood cells, and a final elution in 50 μL instead of 100 μ L was made to obtain higher DNA concentration. The final DNA concentration was assessed by spectrometry and adjusted to 25 ng.μL^-1^.

Genotyping was performed with 10 microsatellite loci [25,48,49]. PCR was conducted using MasterMix(r) (Qiagen) premixed TAQ-polymerase, dNTPs and MgCL_2_, in a total reaction volume of 12.5 μL (6.75 μL MasterMix, 2 μL of each primer pair, 0.5 μL DNA, H_2_O up to a final volume of 12.5 μL). Amplification followed a touchdown protocol, with a 5-minute denaturation at 95°C, 12 amplification cycles of a 30 second denaturation at 95°C, 30 seconds of annealing beginning at 63°C in the first cycle and decreasing one degree at each cycle until 52°C, and 30 seconds of elongation at 72°C, followed by 23 cycles of 30 seconds at 95°C, 30 seconds at 52°C, and 30 seconds at 72°C. Amplification was finished with 10 minutes at 72°C. The same protocol was used for all primers. FAM and HEX fluorochrome-labelled primers were used for sequencing: several microsatellite loci could be multiplexed in the same reaction (Table 1). When multiplex amplification failed, samples were amplified at each locus separately, and pooled together for electrophoresis. Sequencing was performed on an ABI3730 capillary sequencer. 1 μL of PCR product was diluted in 9 μL H2O, and 1 μL of this solution was added to 10 μL formamide and 0.2 μL GeneScan 500-ROX size standard (Applied Biosystems). Output files were analysed on GeneMapper (Applied Biosystems). A random 10% of the samples were amplified and genotyped a second time to check the repeatability and accuracy of the readings.

**Table 1:** Microsatellite analysis: multiplexing parameters and summary statistics.

	Ech030		Ech036		Ech051		Ech071		AM13		B3–2		Emm4		RM3	
PCR	Multi_A		Multi_A		Multi_B		Multi_B		Single		Multi_C		Single		Multi_C	
Genotyping	Seq_A		Seq_A		Seq_B		Seq_B		Seq_C		Seq_C		Seq_A		Seq_C	
Label	HEX		HEX		FAM		FAM		HEX		HEX		FAM		FAM	
Pattern	(CTAT)_*n*_		(GT)_*n*_		(GT)_*n*_		(CTCAT)_*n*_		(GT)_*n*_		(GT)_*n*_		(CT)_*n*_		(CA)_*n*_	
Allele count	9		8		8		13		6		9		9		4	
bGene diversity	0.773		0.750		0.544		0.866		0.702		0.642		0.658		0.541	
Allelic richness	8.988		7.994		7.988		12.994		6.000		8.994		9.000		4.000	
	Allele	Freq.	Allele	Freq.	Allele	Freq.	Allele	Freq.	Allele	Freq.	Allele	Freq.	Allele	Freq.	Allele	Freq.
	267	0.024	190	0.024	188	0.012	215	0.009	109	0.006	285	0.009	233	0.003	202	0.012
	271	0.045	194	0.179	192	0.006	220	0.027	111	0.066	288	0.033	235	0.012	207	0.518
	275	0.232	196	0.003	194	0.580	225	0.051	115	0.326	290	0.009	237	0.407	208	0.438
	279	0.330	198	0.015	196	0.345	230	0.057	117	0.371	292	0.238	239	0.410	210	0.033
	283	0.223	200	0.244	198	0.039	235	0.152	119	0.228	294	0.536	241	0.087	-	-
	287	0.113	202	0.378	200	0.012	240	0.185	121	0.003	296	0.113	243	0.033	-	-
	291	0.027	204	0.125	204	0.003	245	0.176	-	-	298	0.039	245	0.024	-	-
	295	0.003	206	0.033	206	0.003	250	0.176	-	-	300	0.021	247	0.015	-	-
	299	0.003	-	-	-	-	255	0.092	-	-	302	0.003	249	0.009	-	-
	-	-	-	-	-	-	260	0.048	-	-	-	-	-	-	-	-
	-	-	-	-	-	-	265	0.015	-	-	-	-	-	-	-	-
	-	-	-	-	-	-	270	0.012	-	-	-	-	-	-	-	-
	-	-	-	-	-	-	280	0.003	-	-	-	-	-	-	-	-

### Analysis of microsatellite data

Hardy-Weinberg equilibrium was tested in *adegenet* [50] with 100,000 permutations, and in Fstat [51]. F-statistics were assessed in Fstat together with microsatellite summary statistics (allele range, count and frequencies, and linkage disequilibrium). Global intrinsic differentiation in the sample, regardless of any spatial grouping, was tested in Structure [52], with 200,000 burn-in and 1,000,000 steps, under Admixture model, with K values ranging from 1 to 6 (allele frequencies correlated). Ln probability of the data was calculated in Structure Harvester [53].

### Spatial autocorrelation analyses

In a population with limited, yet homogeneous, internal dispersal and gene flow, genetic drift is expected to generate a pattern of isolation by distance. Related genotypes will tend to become clustered in spatial patches, and correlation between genotypes will decay with distance [54]. In this case, correlation between genetic and spatial distance between samples should be observed. In order to test that prediction, spatial autocorrelation analysis was performed across the whole sampling area using Genalex [55], through multivariate analysis of pairwise euclidean and square genetic distances, regardless of any spatial grouping. Significance of autocorrelation was assessed through random permutation of samples across the whole area, and subsequent bootstrapping of the samples contained within pre-defined distance classes around each individual. Permutation tests are used to assess the randomness of the distribution, while bootstrapping allows for the evaluation of the weight of potential individual outliers. We used distance classes starting at 2 meters (the average minimum distance between two sampled nests), and no longer than 8 meters (the average radius of our clusters). Longer distance classes were excluded as they would give too much weight to cross-cluster comparisons. 100,000 random permutations were performed.

However, heterogeneous spatial processes may not always be detected by global spatial autocorrelation analyses [56]. Heterogeneous fine-scale genetic structure was therefore assessed in Genalex using the 2D-Local Spatial Analysis algorithm (2D-LSA, [36]). Each individual was tested for genetic relatedness to its *n* nearest neighbours in order to identify fine-scale patches of lower genetic diversity. This method differs from standard autocorrelation tests in that it completely removes further distance classes from the analysis, and gives the same weight to all individuals considered as neighbours. Thus, it takes into account the possibility of locally restricted areas, within which all individuals are equivalently related and therefore do not exhibit spatial autocorrelation. Once again, significance levels were estimated through random permutation of the samples. Analyses were performed with a number of neighbours ranging from 8 to 10 (the approximate closest 5% of the colony), and 100,000 random permutations.

### Inbreeding and relatedness

Individual inbreeding coefficients and pairwise relatedness were also taken as an index of local genetic structure. If potential mates are more related to each other in some areas, then offspring sampled in that area are expected to be more inbred, and potentially related to each other. Inbreeding was studied both at population and individual levels. At population level, Ballou’s maximum likelihood method was preferred [57], since it has been shown to better estimate population parameters provided sample is large enough [58]. However, MLE methods are known to be sensitive to small sample size, and they are therefore less suitable for estimating individual parameters. Individual inbreeding and relatedness were thus calculated using Ritland’s method-of-moments estimators [59]. Ballou’s MLE was calculated in *adegenet* [50]. Individual means were extracted from a subset of 2,000 values sampled from 20,000 estimates, and were then averaged across the whole sample. Ritland’s MME was computed in *Coancestry* [60]. As expectations for individual inbreeding levels vary and cannot be reliably imported from another study organism, expected inbreeding distribution was estimated through simulation. A virtual population of entirely non-related individuals (as is the expectation for a large, ideal and randomly sampled population) was generated with the same allele frequencies and per-locus homozygosity levels as the sampled colony, and empirical data was compared to the simulated individual inbreeding distribution. The difference in average individual inbreeding between the observed and the simulated distribution was assessed through a bootstrapping method as implemented in *Coancestry*: 50 independent simulated datasets were generated. For each of them, both observed and simulated individuals were pooled, and 100,000 randomly redistributed partitions were generated. Group-averaged inbreeding difference was computed for each of these partitions, and significance of the observed result was checked against this random distribution. Observed individual inbreeding was then mapped onto the colony to identify potential high-inbreeding clusters using *Quantum GIS* (Quantum GIS Development Team, 2013).

### Pairwise cluster analysis

If our second hypothesis (heterogeneous structuring processes) holds true, we expect these processes to apply differently in specific areas of the colony depending on local characteristics. We therefore performed the following analyses on the discrete natural clusters defined in the sampling area (Fig. 1), as opposed to the continuous models applied above. Genetic differentiation between clusters was tested in *Genepop* (exact G-test, default Markov chain parameter values) and *Fstat* using chi-square assessment of genotypic frequencies. Pairwise divergence in inbreeding and relatedness distribution between clusters was also assessed. Pairs of individuals belonging to one cluster were compared to pairs of individuals belonging to different clusters, and significance of distribution divergence was tested in *Coancestry* (10,000 bootstrapping samples).

### Site-quality descriptors analysis

To measure breeding site quality, several ecological variables were used as proxies. *(i)* For 3 consecutive breeding seasons (2010–2012), chick survival rate between hatching (January/February *t*) and fledging (November/December *t*) was measured as the proportion of marked chicks still alive at fledging [61]. *(ii)* Parasite infestation has been shown to influence breeding success and adult survival rate in king penguins [62]. Thus, for 8 consecutive years (2005–2012), tick infestation was measured as the proportion of infested adults in a randomly-selected sample (N = 50) within each colony grid zone. *(iii)* Finally, as early breeders lay eggs in November/December, and late breeders after January [63,64], an estimate of the ratio of incubating and brooding birds in early March was used, as preferred nesting sites are expected to be occupied first by early breeders, while late breeders would occupy remaining, lower-quality areas. For 8 consecutive years (2006–2013), site occupancy timing was thus indexed as the proportion of brooding birds among 50 randomly-chosen breeders (i.e. incubating or brooding birds, leaving non-breeders aside). This results in a score of 1 for preferred early-breeding sites, and a score of 0 for locations only occupied by late breeders.

Correlation between successive years per unit of space was assessed for all 3 variables (Pearson’s *r* test performed between successive years with all grid zones), and spatial autocorrelation of the data was assessed in *spdep* ([65], Moran’s I, two-sided test, using the closest 5%, or 8 nearest neighbours). Our underlying hypothesis being that the breeding-site location choice of an individual breeder depends on its long-term experience and knowledge of breeding-site quality, ecological data were then concatenated for all available sampling years on each space unit. Adult tick infestation on each grid-location was averaged across all 8 sampling years. Chick survival rate was spatially averaged through local spatial analysis, meaning that each sampling location was weighted with the mean survival of the closest 5% of the colony (8 nearest sampling sites) within each breeding year, and data for all years were simultaneously mapped onto the colony. Areas with generally high score have therefore a consistently high local survival rate across several breeding seasons. Spatial autocorrelation within each dataset was assessed through Moran’s I test implemented in *spdep* R package [65], with the closest 5% of the colony defining the spatial weights. For representation purposes, each nesting location was attributed its local tick load, chick survival score and occupancy score, and values were interpolated across the whole colony in Quantum GIS, using squared inverse distance algorithm.

Finally, in order to assess the relation and overlap of the 3 included ecological variables, we performed a principal component analysis (as implemented in R package FactoMineR [66]). Each variable was averaged by grid zone, across all available years, for the peripheral areas where the sampling was conducted. The first principal component was plotted on the colony using Quantum GIS.

## Results

### Microsatellite data

Of the 10 microsatellite loci tested for genotyping, only 8 were successfully amplified, and were used in the subsequent analyses. Ech011 amplified irregularly in king penguins under the tested PCR conditions, and Ech081 was repeatedly scored as more than 2 alleles per genotype. Only Ech030, Ech036, Ech051, Ech071, AM13, B3–2, Emm4 and RM3 were therefore retained for genotyping. For these, scoring repeatability was of 100% across our random replicates.

The total population was found to be in Hardy-Weinberg equilibrium. After 100,000 randomisations, no locus showed significant departure from Hardy-Weinberg equilibrium (*P-values*: Ech030 = 1; Ech036 = 0.9997; Ech051 = 0.3880; Ech071 = 1; AM13 = 1; B3–2 = 0.4948; Emm4 = 1; RM3 = 0.0697). *F*_*IT*_ was not found to be significantly different from zero for the whole sample (*F*_*IT*_ = 0.0259), although it was significantly higher for one locus (Ech071: *F*_*IT*_ = 0.1275). Considering this might be a sign of weak linkage to a region under selective pressure, subsequent analyses were therefore tested both including and excluding this locus. No locus was found to be under linkage disequilibrium. Allele frequencies, counts, and genetic diversity are given in Table 1.

**Fig 2:**
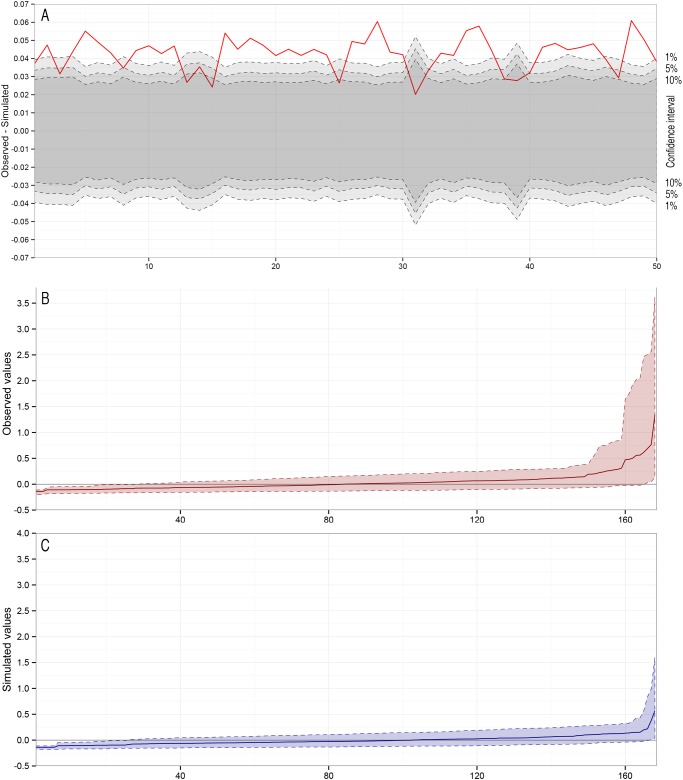
Observed individual inbreeding distribution deviates from expected distribution in a non-related sample. (A) Difference between simulated and observed mean population inbreeding level (Ritland’s coefficient) for 50 simulated datasets. Solid red line: observed difference. Gray intervals: 1%, 5% and 10% confidence intervals. (B) Observed distribution in the sample. (C) Expected distribution in a non-related population, given the same parameters as the observed population.

**Fig 3:**
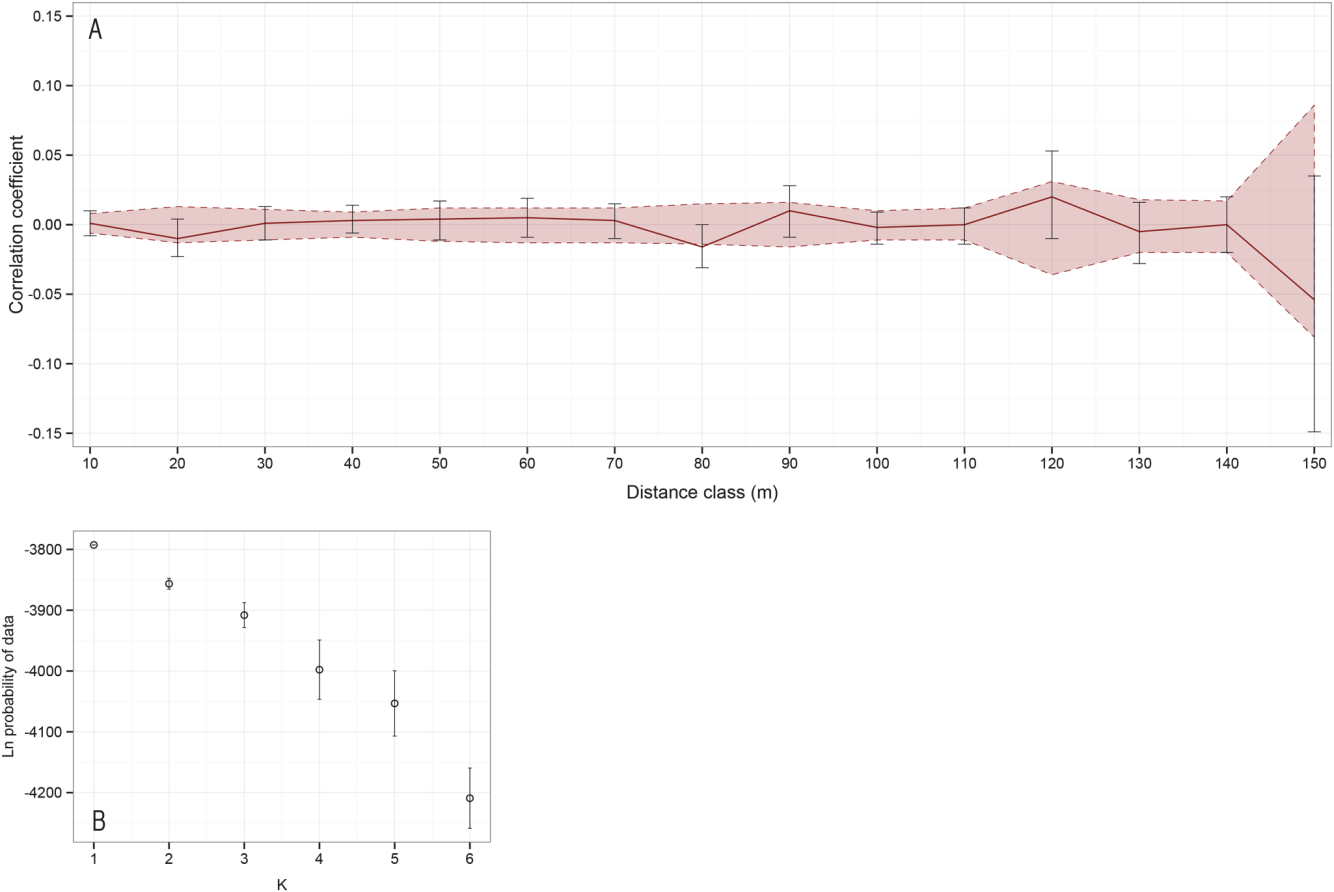
Individual inbreeding and nearest-neighbours-relatedness tend to have a clustered distribution throughout the colony. (A) Distribution of Ritland’s individual inbreeding coefficient along the sampling area. Shaded zones represent analysis clusters C1 to C6. Gray circles signal point displacement for representation purposes. (B) 2D-LSA scores (9 neighbours analysis). Red triangles represent individuals that are significantly more related to their 9 nearest neighbours than to random individuals.

Population inbreeding indicator mean value across our sample was *F* = 0.2321 ± 0.0958 (Ballou’s MLE, standard error: 0.0074). At the individual level, MME inbreeding coefficients were found to deviate from a random distribution. In 80% of our simulations, the empirical data fell outside the 95% interval of the random distribution, and in all cases the difference was clearly positive (empirical mean inbreeding clearly higher than simulated, Fig. 2a). In particular, the lower (outbred) range was strongly under-represented as compared to a random, non-related sample as simulated in *Coancestry* (Fig. 2b-c). Ritland’s inbreeding coefficient was then mapped on the colony. Two more inbred regions appeared, at the south-east and in the centre of the sampling area (Fig. 3a), corresponding to clusters C3 and C5–C6.

### Spatial analysis

**Fig 4:**
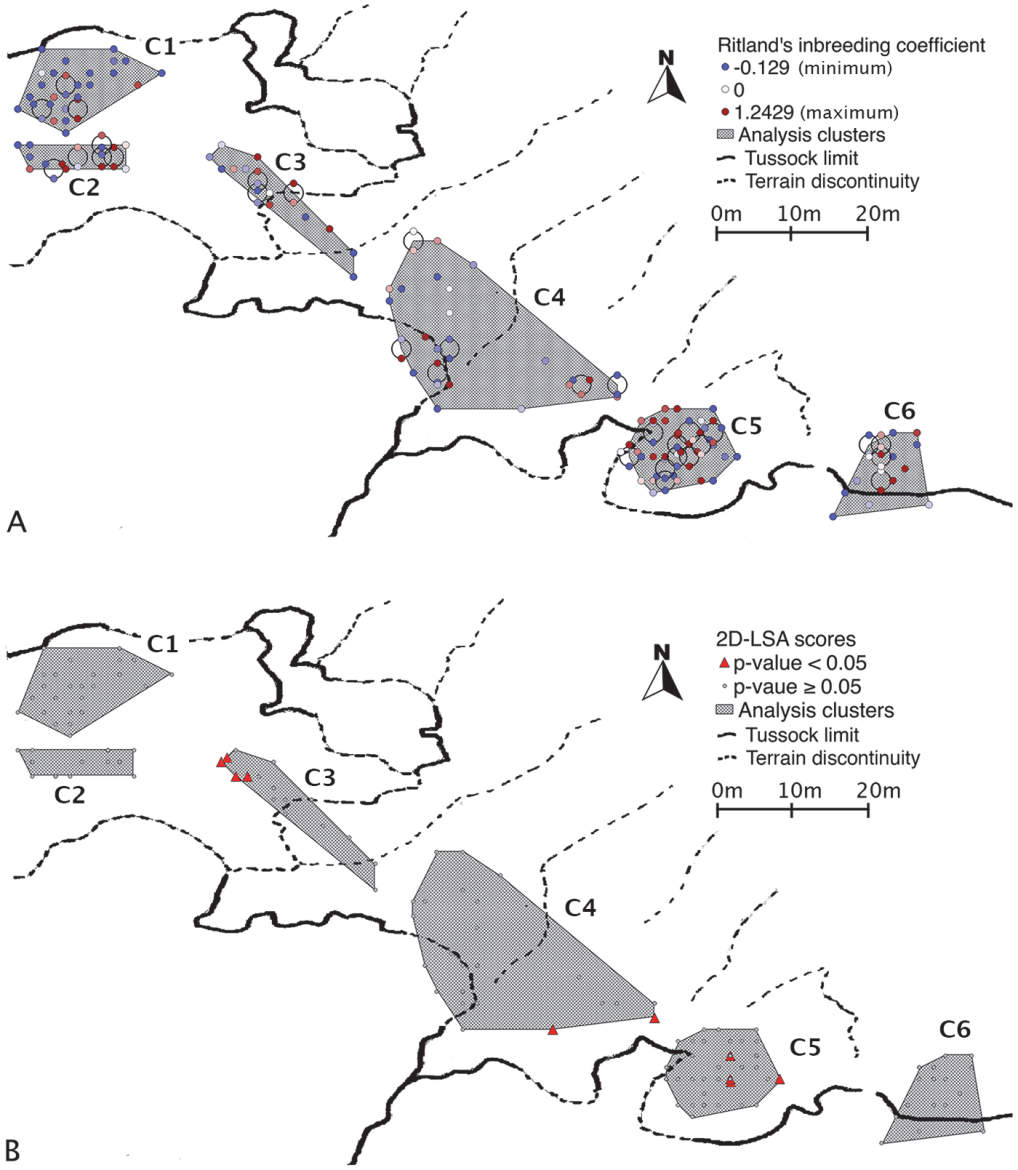
Spatial correlation and intrinsic clustering tests do not support strong structure. (A) Spatial correlogram. Solid red line: observed correlation, by distance classes. Dashed red lines: random expectation confidence interval, determined by bootstrapping. (B) Ln probability of the genetic data under six different clustering situations (from 1 to 6 populations).

Spatial autocorrelation tests performed on the whole population in *Genalex* did not yield any significant structure across the sampling area (Fig. 4a), suggesting no visible decay in correlation in the first distance classes, as would be expected if genetic diversity varied along a gradient across the colony. Intrinsic clustering of the dataset, assessed in *Structure*, did not yield any significant results either (Fig. 4b). In our 2D-LSA analyses however, 10 individuals across our sample were found to have significant higher genetic correlation with their nearest 5% of the colony (9 individuals) (*P* < 0.05). It is noteworthy that these individuals were clustered in two groups that strongly overlapped with the higher-inbreeding regions identified previously (clusters C3 and C5, see Fig. 3b). Genetic correlation decayed quickly when we increased the number of neighbours used in the analysis above the nearest 5% of the colony.

Cluster differentiation analyses performed in *Genepop* showed that cluster C3 was significantly different from 3 other clusters (genotypic differentiation, exact *G*-test: *P*_C1_ = 0.0277, *P*_C4_ = 0.0326, and *P*_C5_ = 0.0268). However, pairwise *F*_*ST*_ comparisons did not yield any significant result.

Heterogeneity in inbreeding distribution across different clusters was tested pairwise in *Coancestry* (bootstrapping: 10,000): cluster C1 was found to have significantly (*P*_C1_ < 0.05) less inbreeding than clusters C2, C3, C5 and C6 (but not C4). Most significant was the comparison between C1 and C5 (Table 2). In the same way, pairwise individual relatedness was found to be significantly higher in C6 than in C1 and C4 (other pairwise comparisons were non-significant, see Table 2).

**Table 2:** Pairwise cluster comparison of inbreeding and relatedness distributions reveal genetic heterogeneity in the sampling area.

	Cluster 1		Cluster 2		Cluster 3		Cluster 4		Cluster 5		Cluster 6	
												
			**-1.1002E-001**		**-1.5355E-001**		**-3.9188E-002**		**-7.0628E-002**		**-6.3993E-002**	
**Cluster 1**			*8.4695E-002*	C1<C2*	*1.0299E-001*	C1<C3*	*4.0492E-002*	C1 = C4	*5.2465E-002*	C1<C5*	*5.8778E-002*	C1<C6*
			*-8.5559E-002*		*-1.2204E-001*		*-4.3607E-002*		*-5.1926E-002*		*-5.8408E-002*	
												
	**1.0118E-002**				**-4.3531E-002**		**7.0834E-002**		**3.9394E-002**		**4.6029E-002**	
**Cluster 2**	2.0834E-002	C1 = C2			*1.5727E-001*	C2 = C3	*7.9085E-002*	C2 = C4	*8.1326E-002*	C2 = C5	*1.0041E-001*	C2 = C6
	*-2.3307E-002*				*-1.6080E-001*		*-7.1760E-002*		*-7.3237E-002*		*-9.9416E-002*	
												
	**-4.0898E-003**		**-1.4208E-002**				**1.1436E-001**		**8.2925E-002**		**8.9560E-002**	
**Cluster 3**	*2.1038E-002*	C1 = C3	*3.2650E-002*	C2 = C3			*1.1821E-001*	C3 = C4	*1.1181E-001*	C3 = C5	*1.4201E-001*	C3 = C6
	*-2.3405E-002*		*-3.4437E-002*				*-8.6573E-002*		*-8.5461E-002*		*-1.2877E-001*	
												
	**4.5949E-003**		**-5.5231E-003**		**8.6848E-003**				**-3.1439E-002**		**-2.4804E-002**	
**Cluster 4**	*1.1684E-002*	C1 = C4	*1.9016E-002*	C2 = C4	*-1.7182E-002*	C3 = C4			*4.7974E-002*	C4 = C5	*4.7573E-002*	C4 = C6
	*-1.1589E-002*		*-1.6720E-002*		*1.7959E-002*				*-4.4700E-002*		*-5.3248E-002*	
												
	**8.1903E-003**		**-1.9277E-003**		**1.2280E-002**		**3.5953E-003**				**6.6350E-003**	
**Cluster 5**	*1.4813E-002*	C1 = C5	*2.4833E-002*	C2 = C5	*2.7328E-002*	C3 = C5	*1.2583E-002*	C4 = C5			*5.8991E-002*	C5 = C6
	*-1.3828E-002*		*-2.0848E-002*		*-2.2622E-002*		*-1.1928E-002*				*-6.3224E-002*	
												
	**1.8131E-002**		**8.0130E-003**		**2.2221E-002**		**1.3536E-002**		**9.9407E-003**			
**Cluster 6**	*1.6586E-002*	C1<C6*	*2.6127E-002*	C2 = C6	*2.3997E-002*	C3 = C6	*1.3248E-002*	C4<C6*	*1.8263E-002*	C5 = C6		
	*-1.7630E-002*		*-2.4755E-002*		*-2.3723E-002*		*-1.3465E-002*		*-2.1122E-002*			
												

Upper triangle: Ritland’s individual inbreeding coefficient. Lower triangle: Ritland’s pairwise relatedness coefficient. The empirical coefficient is shown in bold, followed by the 95% confidence interval. Significantly divergent comparisons are shown in bold type with asterisks.

**Fig 5:**
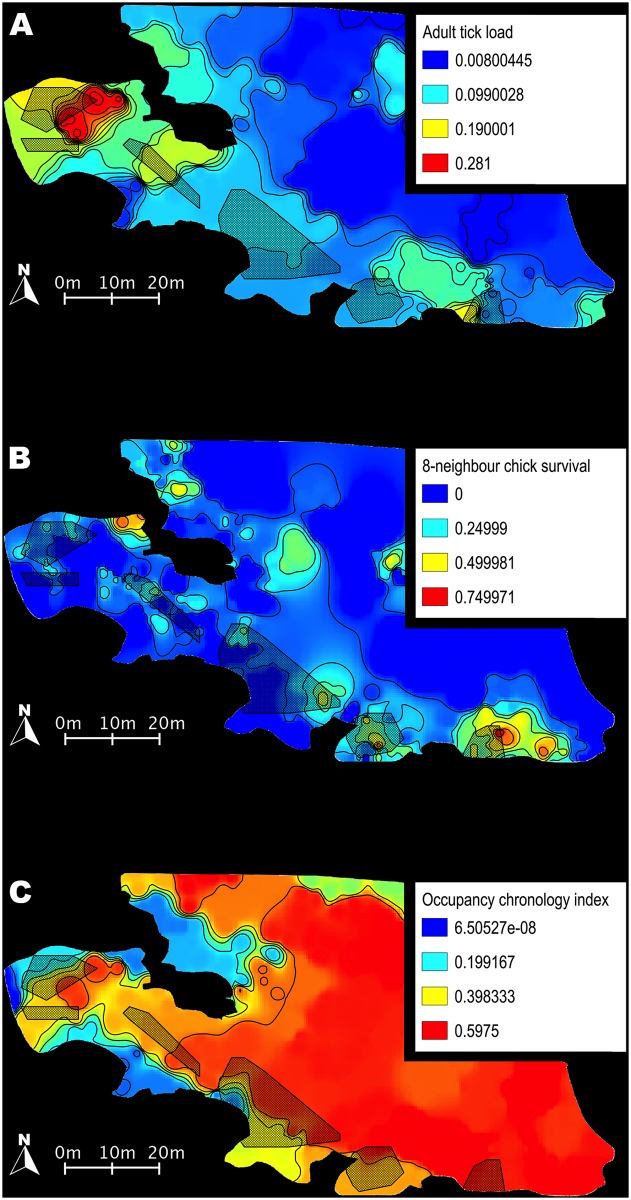
Ecological descriptors of breeding-site quality exhibit a strongly heterogeneous distribution across the colony. (A) Adult tick load, averaged for years 2005–2012. Contour lines: 0.02 steps. (B) 8-neighbour chick survival, averaged for years 2010–2012. Contour lines: 0.1 steps. (C) Site occupancy chronology, averaged for years 2006–2013. Ratio of brooding birds amongst 50 randomly selected breeders. Contour lines: 0.1 steps.

**Fig 6:**
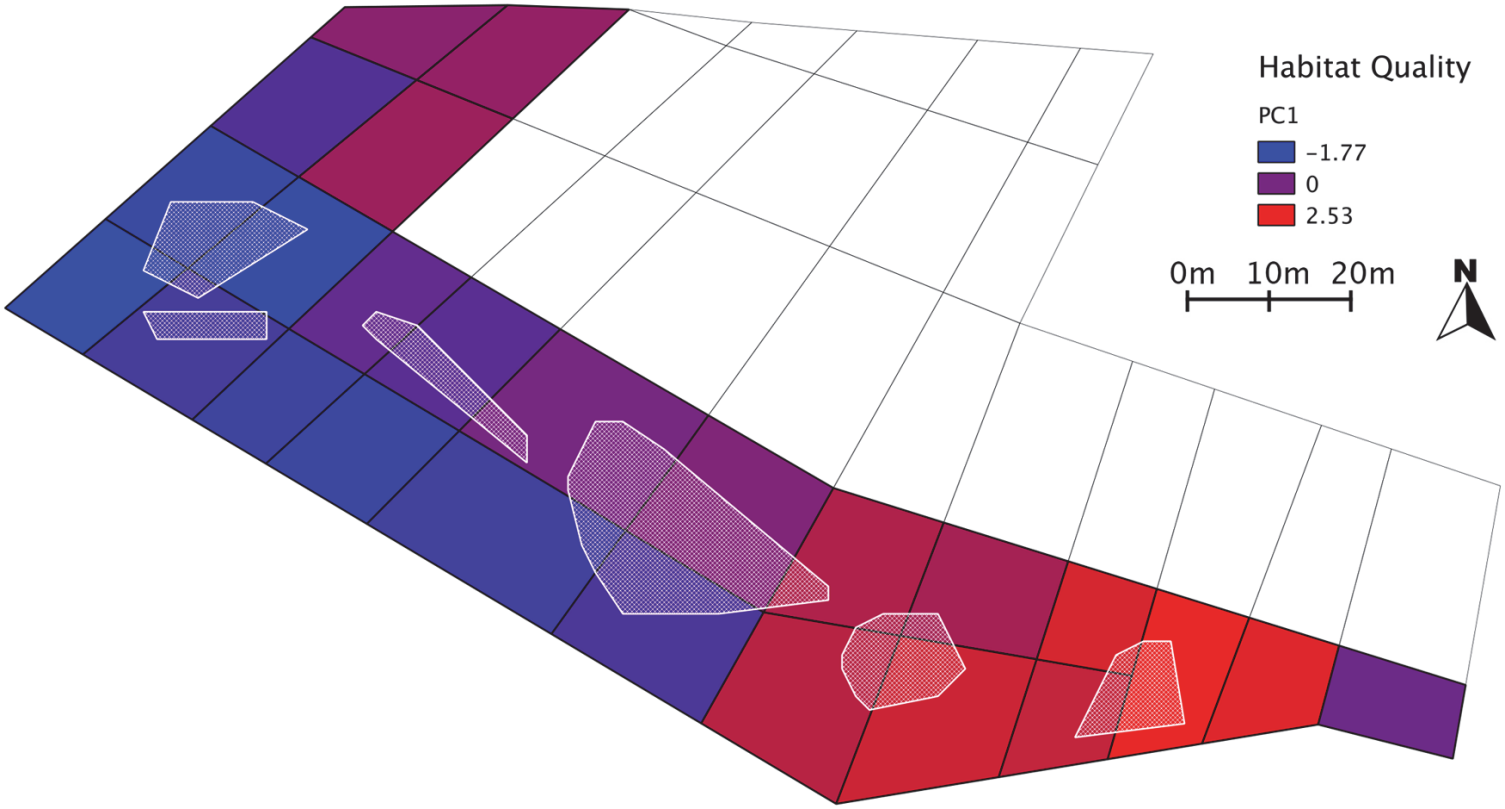
First axis of principal component analysis as a summary habitat-quality index. White polygons: clusters C1 to C6.

Our analyses revealed strong overlap between this clustering pattern and spatial distribution of ecological site-quality indicators. Tick infestation and site occupancy timing showed strong year-to-year correlation across all 8 sampled years (slope c and significance level of the correlation *P* for tick load from one year to the previous one,: 2004: c = 0.400 *P* ≈ 0; 2005: c = 0.465 *P* ≈ 0; 2006: c = 0.146 *P* < 0.05; 2007: c = 0.406 *P* ≈ 0; 2008: c = 0.577 *P* < 0.001; 2009: c = 0.424 *P* < 0.01; 2010: c = 0.192 *P* < 0.01; 2011: c = 0.743 *P* ≈ 0. For site occupancy timing from each year to the previous one, 2007: c = 0.526 *P* ≈ 0; 2008: c = 0.712 *P* ≈ 0; 2009: c = 0.462 *P* ≈ 0; 2010: c = 0.664 *P* ≈ 0; 2012: c = 0.159 *P* < 0.01; 2013: c = 0.010 NS. Many missing data in 2011 led us to discard this year, and to correlate 2012 values with 2010). Chick survival was found to be correlated between 2010 and 2011 (c = 1.548 *P* = 0.0427), sampling did not overlap sufficiently between years 2011 and 2012 for comparison. All 3 variables showed a markedly structured, gradient-driven distribution across our sampling range [65], and were found to be strongly autocorrelated (Moran’s I, two-sided test, using the closest 5%, or 8 nearest neighbours, all *P*-values < 0.01). Occupancy timing and chick survival distributions appeared to largely overlap, and to be partly complementary to tick infestation distribution. Comparison between C1 and C5 was significant for all variables: tick infestation varied from 0.188 ± 0.095 in C1 to 0.083 ± 0.019 in C5, while averaged chick survival ranged from 0.208 ± 0.058 in C1 to 0.398 ± 0.130 in C5, and occupancy timing index from 0.303 ± 0.206 in C1 to 0.488 ± 0.056 in C5 (Fig. 5). We did not find any correlations between the monitored habitat-quality descriptors and the individual-level or cluster-level genetic descriptors. However, when running a principal component analysis including the 3 ecological variables, the south-eastern end of the colony (higher habitat quality) and the north-western end (lower habitat quality) were clearly separated on the first axis, which accounted for 40.5% of the total variation (correlation coefficients, contributions: chick survival 0.82, 55.6%, occupancy timing 0.62, 31.6%, tick load-0.39, 12.8%) (Fig. 6).

## Discussion

### A near-panmictic population

Our data on colony-wide spatial structure contradict the hypothesis of a strong, homogeneous process shaping the genetic structure of our studied king penguin sub-population. Hardy-Weinberg equilibrium held across all loci, population-wide *F*_*IT*_ was not significantly skewed [39,67], and spatial autocorrelation tests performed across the whole sampling area were non-significant [35,54]. Analyses of intrinsic partitioning indeed systematically pointed to a non-structured population [68]. Although it is indeed possible that the limited number of markers used explains part of that lack of differenciation signal, microsatellite loci are known to have a high mutation rate in spheniciforms [69] and generally enough polymorphism to allow for fine-scale genetic comparisons and structure detection, even within a population [70].

Tests of genotypic differentiation did yield significant results for cluster C3, however this finding is subject to caution as it is not confirmed by pairwise *Fst* comparisons. In fact, G-test has been shown to often be over-sensitive [71], and to yield numerous false positives [72]. These findings allow us to reject strong underlying structure resulting from constant, homogeneous processes such as multi-generation isolation of sub-colony units. Yet, in wild populations, a lack of global visible genetic structure does not necessarily imply the absence of structuring processes [38]. Indeed, spatial structure may evolve as a secondary dependency to heterogeneous habitat features [56].

### Heterogeneity in inbreeding probability

Our investigations of inbreeding probability highlight the fact that analyses taking into account possible heterogeneity in structuring processes do point towards a non-panmictic population. Observed population inbreeding descriptor *F* = 0.231 is remarkably high—it implies a homozygosity level close to what is expected in a half-sibling relatedness context, for example [57,73]. Although homozygosity-based inbreeding calculations do not necessarily imply true (i.e. pedigree-deduced [74]) inbreeding [75], our result shows a strong bias in the mating system, resulting in non-random gene mixing. This interpretation is supported by the individual inbreeding distribution, which significantly deviates from an equivalent random distribution, with outbred classes being strongly under-represented as compared to a non-related group of individuals [74]. Moreover, more inbred individuals tend to be concentrated at the south-eastern end of the sampling range of our sub-colony (clusters C5 and C6), while more outbred individuals are more frequent at the north-western end (cluster C1). Pairwise relatedness within spatially delimited areas has also been shown to reflect weak genetic structure in wild populations [37]. Not surprisingly, we found that individuals that are significantly related to their direct neighbours tend to cluster in areas that largely overlap more inbred areas.

### Pairwise cluster differentiation

Our pairwise cluster analysis supports the hypothesis of genetic clustering within the colony. Genotypic differentiation points towards structural divergence between the centre and the extremities of our sampling range. Yet, the genotypic structure detected is globally weak. This is an expected result, as Waples and Gaggiotti [76] showed that microsatellite-based genotypic cluster divergence faded out when movement among clusters exceeded 10%, which is likely the case in seabird colonies (e.g. [28]). More importantly, the north-western cluster (C1) appeared significantly less inbred than any other cluster. Similarly, C1 and C4 both showed low internal pairwise relatedness levels. This trend was not marked enough to be significant throughout all pairwise comparisons (comparison between C1 and C5, for instance, was near-significant).

Together, these different elements outline an underlying structure in which the north-western end of the colony (cluster C1 in particular) appears more outbred, with lower pairwise-relatedness levels, and the south-eastern end (especially cluster C5) is more inbred, with higher relatedness levels. Such a structure implies that the probability for a breeder to mate with a related individual is not constant across the colony. Potential mates are therefore more likely to share identical alleles (either by descent or by state) at the south-eastern end of the colony than at the north-western end. This result is in accordance with the findings of Steiner and Gaston [28], who showed, from direct observations, that, in colonial seabirds, even a very short-distance intra-colony dispersal may significantly impact breeding success and inbreeding avoidance.

### Heterogeneity in breeding site quality

Interestingly, the observed heterogeneity in genetic structure we found within this king penguin colony appeared to be related to the quality of the breeding sites. Tick infestation has a local maximum around C1, i.e. at the north-western end of the range, and lower values elsewhere. In contrast, chick survival has local maxima around C5 and C6, and very low values in C1. The chronology of settlement-site occupancy also appeared to be strongly associated with the quality of the breeding sites. Indeed, preferred areas at the south-eastern end of the colony were occupied first, and less-favoured spots at the north-western end were occupied by late breeders. The fact that no significant correlation could be found between habitat descriptors and genetic parameters may be a consequence of our small sample size, as our sampling area is not large enough to allow for replicate situations. Some other constraints, such as flooding, fine-scale variations in predation pressure, or inter-annual climate variability, were not monitored and may also interact to define high- and low-quality habitat.

Overall, principal component analysis revealed a gradient from higher habitat quality at the south-eastern end of the colony, around clusters C5 and C6, to lower quality at the north-western end (clusters C1 and C2). This pattern suggests that habitat quality may be one of the drivers shaping the observed differences in relatedness and inbreeding across sampled individuals. This may be a consequence of differences in fine-scale philopatric behaviour. Indeed, while king penguins have been shown to select their breeding location according to site-specific characteristics at the breeding-season scale [30], our results suggest that breeding experience may drive settlement site choice across multiple generations, and that this process may be widespread enough to impact genetic patterns in the colony.

### Effects of life-history and demography

Peripheral territories in a king penguin colony are generally characterized by higher tick loads and later settling dates compared to central areas, thus the weak genetic signal may be explained by our peripheral sampling design (required in order to minimize colony disturbance). Nevertheless, central areas do suffer from specific pressures, such as higher density and associated stress levels [77]. Predation pressure, on the other hand, is not expected to differentiate peripheral and central areas. Although it has been shown that immediately peripheral birds spend twice as much time interacting with predators than central birds [78], most chick mortality has been shown to happen during winter, especially through predation by giant petrels (*Macronectes spp*.) {[79], when chicks have gathered in crèches. It has been shown [80] that successful predation events during this time, either by giant petrels or brown skuas (*Stercorarius antarcticus*), are not more common in the peripheral areas than in the centre of the colony, which is consistent with our field observations. Finally, the overall breeding success has been shown to be independent of central or peripheral location [81], which suggests that purely geometric location in relation to the colony (such as centre-to-periphery gradients) is not the main determinant of habitat quality. We can therefore assume that spatial processes driving breeding site choice identified on the edges of a colony should also apply at its centre.

Another possible explanation of the weakness in the genetic signal may arise from our sampling of pre-dispersal chicks and the analysise of nuclear autosomal markers (as opposed to sex-specific ones). Indeed, a constant bias such as sex-specific dispersal may lead to more genetic structure in one sex than the other, a phenomenon which may be blurred by our sampling of pre-dispersal individuals of undetermined sex. Yet, although sex-specific dispersal is a common phenomenon in birds [82], on a multi-generation scale, it is only expected to influence the structure of sex-specific markers, and not of autosomal loci, which are subject to admixture [83]. Thus, our sampling design should only reduce our ability to detect instantaneous sex-specific phenomenon, and not population-wide, sex-independent structures.

A more likely explanation of the weak genetic signal may lie in inter-annual habitat variability. If local philopatric maxima are conditioned by the past breeding experience of individuals, they may be subject to medium time-scale variations: higher-quality breeding sites may change every few generations, depending on flooding variations, ground erosion, or parasite pressures. These very fine-scale shifts may be sufficient to weaken genetic structure at the colony level on longer time scales.

Moreover, at the individual scale, it may be difficult for a breeder to retain its breeding site consistently across all breeding seasons. Indeed, the complex breeding cycle of the king penguin extends for more than a year, implying that birds are alternately early or late breeder [31,47], regardless of individual quality [84]. Therefore, even experienced breeders might sometimes be forced to settle in lower-quality spots due to site-occupancy contingency at the season-level, and the output of their previous breeding season. This process could maintain a sufficient rate of genetic mixing in the whole colony despite a general trend towards local structure.

Finally, king penguin population genetic structure may still bear the imprint of recent demographic history. Most of the king penguin colonies suffered a drastic reduction at the turn of the nineteenth century, as sealers slaughtered them to near-extinction on most of the archipelagos for oil production. They recovered, but recently [85,86]. Similar colonies on Macquarie island were reportedly reduced to less than 1,600 breeding pairs in 1937 [87]. Rapid recovery followed the cessation of slaughtering [45,85], and population growth rate is suggested to have neared intrinsic growth rate for three decades [45]. This complex demographic history is also expected to have had an impact on colonial genetic patterns. Even under the assumption of strong, consistent processes driving structure across several generations, recent rapid growth may weaken a long-term signal in present-day colonies [76], though in that case, the use of more markers or more individuals may increase the strength of the signal [8].

### Heterogeneity as a mixing mechanism

The convergence of genetic and ecological indicators we observed outlines the structural heterogeneity inherent to penguin colonies, and probably to other colonial seabirds (such as guillemots or eiders, [18,88]). However, our data point towards generally transient processes, which are not homogenous and stable enough in time to impact colony structure on a large spatial and temporal frame. Local heterogeneity appears to be dependent on medium-scale factors, such as individual experience and breeding history, but also present-day site quality, and should therefore be considered as a fundamental feature of coloniality in seabirds. Local philopatric hotspots would therefore be counterbalanced by active outbreeding regions, thus preserving global genetic diversity and mixing at the colony level. Instead of being an exception in an otherwise strongly philopatric system, we therefore believe that these lower-quality, strongly outbred areas are playing an active role in mitigating the potentially drastic effects of strong natal philopatry on local genetic drift and loss of diversity. Our findings illustrate how individual life-history decisions, such as site-fidelity or dispersion, are related at the colony level with local environment features. These constitute true colony processes that actively enhance higher-scale population functions, such as genetic mixing and inbreeding avoidance, thereby allowing the persistance over time of philopatric colonial systems.
